# Association of incremental pulse wave velocity with cardiometabolic risk factors

**DOI:** 10.1038/s41598-021-94723-2

**Published:** 2021-07-29

**Authors:** P. M. Nabeel, Dinu S. Chandran, Prabhdeep Kaur, Sadagopan Thanikachalam, Mohanasankar Sivaprakasam, Jayaraj Joseph

**Affiliations:** 1 Healthcare Technology Innovation Centre, IIT Madras, Chennai, 600113 India; 2 Department of Physiology, All India Institute of Medical Sciences, New Delhi, 110029 India; 3 National Institute of Epidemiology, Indian Council of Medical Research, Chennai, 600077 India; 4 Sri Ramachandra Institute of Higher Education and Research, Chennai, 600116 India; 5 Department of Electrical Engineering, Indian Institute of Technology Madras, Chennai, 600036 India

**Keywords:** Biomedical engineering, Prognostic markers, Risk factors

## Abstract

We investigate the association of incremental pulse wave velocity (ΔC; the change in pulse wave velocity over a cardiac cycle) with cardiometabolic risk factors and report the first and (currently) the largest population-level data. In a cross-sectional study performed in a cohort of 1373 general population participants, ΔC was measured using clinically validated ARTSENS devices. There were 455 participants in the metabolic syndrome (MetS) group whose average ΔC was ~ 28.4% higher than that of the non-metabolic syndrome (Non-MetS) group. Females with MetS showed ~ 10.9% elevated average ΔC compared to males of the Non-MetS group. As the number of risk factors increased from 0 to 5, the average ΔC escalated by ~ 55% (1.50 ± 0.52 m/s to 2.33 ± 0.91 m/s). A gradual increase in average ΔC was observed across each decade from the younger (ΔC = 1.53 ± 0.54 m/s) to geriatric (ΔC = 2.34 ± 0.59 m/s) populations. There was also a significant difference in ΔC among the blood pressure categories. Most importantly, ΔC ≥ 1.81 m/s predicted a constellation of ≥ 3 risks with AUC = 0.615, OR = 2.309, and RR = 1.703. All statistical trends remained significant, even after adjusting for covariates. The study provides initial evidence for the potential use of ΔC as a tool for the early detection and screening of vascular dysfunction, which opens up avenues for active clinical and epidemiological studies. Further investigations are encouraged to confirm and establish the causative mechanism for the reported associations.

## Introduction

Cardiovascular disease remains the leading cause of morbidity and mortality, accounting for ~ 31% of all deaths worldwide^1^. The pathogenesis of cardiovascular disease involves a constellation of several risk factors/markers in a single entity that contribute to the development and progression of the disease. Among them, a set of metabolic abnormalities with a common link to the ectopic deposit of lipids, insulin resistance, and chronic low-grade inflammation are the cardiometabolic risk factors^2^. However, early detection of future cardiovascular events using such indirect conventional risk factors is suboptimal and remains uncertain^3^. Therefore, vascular function related parameters are now being investigated as a more direct early marker of subclinical cardiovascular disease^4^. There is a long-standing and considerable evidence base showing that assessment of vascular markers improve risk stratification and optimize the preventive treatment strategy^4^.

Several of the cardiometabolic risk factors are related to structural and functional vascular abnormalities^5,6^. Subsequently, research interest in preventive cardiology has focused on the role of aortic pulse wave velocity (an indicator of arterial stiffness^7^) as a prognostic biomarker of cardiovascular events and multiple risk factors. The most robust and well-studied estimate of pulse wave velocity is the one measured across the carotid-femoral arterial segment^8^. In large population-based studies, carotid-femoral pulse wave velocity has been used to capture the continuum of arterial ageing spanning from healthy vascular ageing on one side to early vascular ageing on the other end of the vascular aging spectrum^9,10^. Studies highlighting the influence of cardiometabolic risk factors on the stiffness of large arteries have also been widely performed with carotid-femoral pulse wave velocity^11–14^. Further, conglomeration of cardiometabolic risk factors has been shown to augment arterial stiffening in patients with chronic kidney disease, cognitive impairment and multiple cardiovascular target organ damage^15–17^.

When pulse wave velocity is measured over a large arterial segment by the traditional method(s)^8^, it provides a single average value per cardiac cycle and ignores the influence of instantaneous arterial pressure fluctuations within each cardiac cycle. More specifically, pulse wave velocity, when measured from the foot of the pulse wave, yields a value at the diastolic pressure level^7^. However, the non-linear elastic nature of large arteries renders their pulse wave velocity to be quasi-periodically variable in accordance with the operating transmural blood pressure throughout a cardiac cycle^7,8^. Its maximum change within a cardiac cycle, from early systole to late systole, is referred to as incremental pulse wave velocity (ΔC)^8^. Analogously, a higher pulsatile increment of arterial blood pressure from diastole to systole has been shown to impact the structure and function of large arteries, including aorta and carotid artery^18^. Since carotid pulse pressure, as opposed to the mean arterial pressure, was observed to have an inverse association with the aortic root diameter, it may be posited that ΔC might provide independent information on dynamic arterial wall characteristics inaccessible otherwise to assessment by routinely used carotid-femoral pulse wave velocity^19^. In this work, we report a non-invasive, direct and clinically feasible method to evaluate ΔC (see “Materials and methods”). Further description of ΔC goes beyond the scope of this article. Interested readers will find a detailed discussion on ΔC and its underlying mechanism in a recent review^8^.

In recent years, studies by independent researchers^20–24^ estimating ΔC from the carotid artery or aorta have explored the clinical significance of ΔC. Remarkably, ΔC has been demonstrated to characterize cardiovascular alterations such as hypertension-derived vascular damage, load-induced myocardial hypertrophy, and Vascular Ehlers-Danlos syndrome above and beyond the conventional risk markers/indices^8^. However, the potential link between ΔC and metabolic abnormalities related to cardiovascular disease has received little attention. Therefore, the present study is aimed to (1) investigate the association between ΔC and cardiometabolic risk factors; (2) investigate population trends of ΔC concerning gender, age, or presence/number of risks; and (3) explore the feasibility of identifying reliable cut-off thresholds of ΔC for risk classification of subjects into either metabolic syndrome (MetS) group with ≥ 3 risks or non-metabolic syndrome (Non-MetS) group with ≤ 2 risks, on the basis of IDF/AHA/NHLBI criteria^2^.

## Results

### Baseline characteristics of the study population

**Figure 1:**
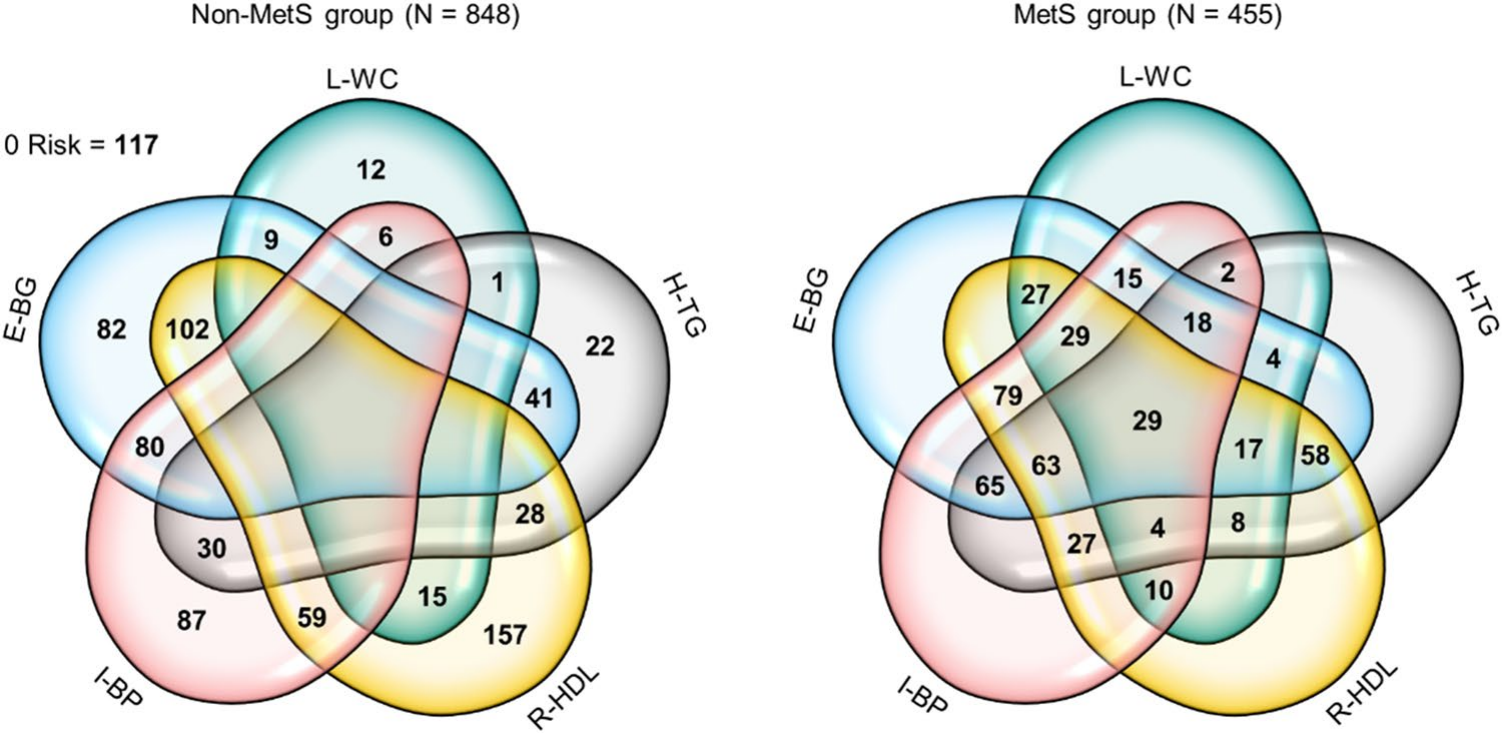
Venn diagrams showing the number of subjects in risk factor combinations of the Non-MetS and MetS groups.

Measured variables were normally distributed. Details on baseline measurements of the subjects who met the inclusion and exclusion criteria are reported in Table 1. Of these 1303 subjects, 455 (308 females and 147 males; age = 41 ± 9.5 years) were identified with MetS (≥ 3 risk factors). There were 381 males and 467 females in the Non-MetS group (age = 40 ± 10 years). Elevated blood glucose (E-BG) was the most prevalent risk factor in the MetS group (88.8%), followed by reduced high-density lipoprotein cholesterol (R-HDL; 77.1%), increased blood pressure (I-BP; 74.9%), high triglycerides (H-TG; 64.8%), and large waist circumference (L-WC; 35.8%). Regardless of the number of risk factors, E-BG, R-HDL, and I-BP were predominant in the study population (Fig. 1). Among the Non-MetS group, subjects with ≤ 2 risk factors (N = 848), 50 females and 67 males were free from all five risks. R-HDL was diagnosed in 43.6% of subjects with only 1 risk factor, and it’s clustering with E-BG and I-BP was diagnosed in 27.5% and 15.9% subjects with 2 risk factors, respectively (Fig. 1).

**Table 1:** Baseline demographics and characteristics of the study population.

Parameter	Number (%)	Value (mean ± SD)
Participants	1303	
Females	775 (59.5%)	
Males	528 (40.5%)	
Age (years)		40.5 ± 10
**Body size measurements**		
Height (cm)		156 ± 8.6
Weight (kg)		58 ± 12.5
Body mass index (kg/m^2^)		24 ± 4.4
Waist circumference (cm)		82.3 ± 11.3
**Biochemical measurements**		
Fasting total triglycerides (mg/dL)		138.16 ± 79.57
Fasting high-density lipoprotein (HDL) cholesterol (mg/dL)		44.99 ± 7.88
Fasting plasma glucose (mg/dL)		113.13 ± 45.43
**Physiological measurements**		
Systolic blood pressure, P_S_ (mmHg)		127 ± 22
Diastolic blood pressure, P_D_ (mmHg)		81 ± 13
Peak systolic (left) carotid lumen diameter, D_S_ (mm)		5.9 ± 1.28
End-diastolic (left) carotid lumen diameter, D_D_ (mm)		5.54 ± 1.26
Incremental pulse wave velocity, ΔC (m/s)		1.79 ± 0.74
**Lifestyle habit**		
Tobacco chewing	139 (10.7%)	
No smoking	923 (70.8%)	
Current smoking	307 (23.6%)	
Past smoking	73 (5.6%)	
No alcohol consumption	936 (71.8%)	
**Clustering of risk factors**		
L-WC	206 (15.8%)	
H-TG	417 (32%)	
R-HDL	712 (54.6%)	
I-BP	603 (46.2%)	
E-BG	718 (55.1%)	
0 risk factor	117 (8.9%)	
1 risk factor	360 (27.6%)	
2 risk factors	371 (28.5%)	
3 risk factors	295 (22.6%)	
4 risk factors	131 (10.1%)	
5 risk factors	29 (2.3%)	
Non-MetS group	848 (65%)	
MetS group	455 (35%)

### Analysis of ΔC for gender, Non-MetS/MetS, and age group

**Figure 2:**
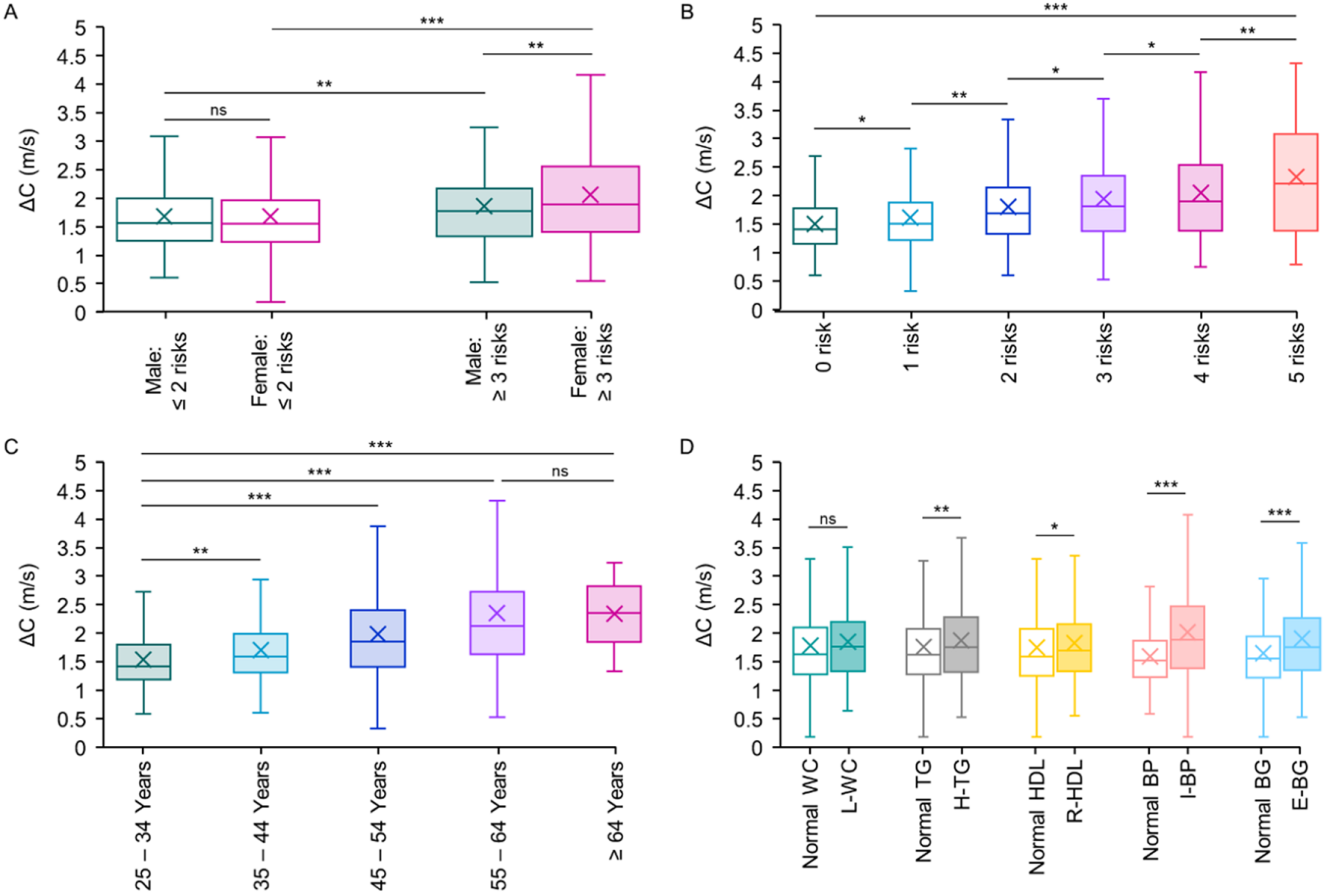
Incremental pulse wave velocity, ΔC, of (**A**) male versus female subjects for Non-MetS and MetS groups, (**B**) Non-MetS and MetS groups with multiple risk factors, (**C**) subjects based on their chronologic age, (**D**) subjects with/without the presence of individual metabolic abnormality (ns p > 0.05, * p ≤ 0.05, ** p ≤ 0.001, *** p ≤ 0.0001).

Associations of ΔC with the age (r^2^ = 0.11, p < 0.0001), mean arterial blood pressure (r^2^ = 0.08, p < 0.0001), and conventional pulse wave velocity (r^2^ = 0.43, p < 0.0001) were observed to be positive. The average ΔC for the Non-MetS and MetS groups were 1.62 ± 0.64 m/s and 2.01 ± 0.86 m/s, respectively. Their between-group difference was statistically significant (p < 0.001) even after adjusting for covariates. In the subset of male subjects with MetS, ΔC averaged to 1.86 ± 0.72 m/s. It was 10.7% higher (p < 0.01) than the average ΔC of Non-MetS male subjects (Fig. 2A). Average ΔC of female subjects with MetS (2.06 ± 0.91 m/s) was 22.6% higher (p < 0.001) than that of the Non-MetS females (Fig. 2A). No significant difference (p > 0.05) was observed between average ΔCs of Non-MetS males and females. Within MetS group, females possessed 10.9% (p < 0.01) elevated average ΔC compared to males’ ΔC (Fig. 2A).

The average age of subjects in each subset (males/females with MetS/Non-MetS) was between 39 and 41 years. After adjustment for covariates, the group average ΔC of MetS male and female subsets were respectively 10.4% and 18.9% higher (p < 0.001) than the corresponding measures of Non-MetS subsets. The adjusted ΔC of MetS females persisted an increased average value (12.6%, p < 0.001) than that of MetS males. When the association of ΔC tested with multiple risk factors, the group average ΔC increased from 1.50 ± 0.52 m/s to 2.33 ± 0.91 m/s (~ 55.0%, p < 0.001) as the number of risks increased from 0 to 5. Note that the average age of subjects in each risk group was between 40–42 years. The percentage increase in ΔC across immediate risk numbers, from 0 to 5 risks, were 7.1%, 12.2%, 7.6%, 5.2% and 14.0% (∀ p < 0.05), respectively (Fig. 2B). All differences remained significant (p < 0.05) after adjusting for covariates (i.e. 7.3%, 12.6%, 5.8%, 5.5% and 13.2%, respectively). The change in ΔC from no-risk to 5-risks was attenuated but persisted (52.6%, p < 0.001) after adjusting for covariates.

Age-specific changes in ΔC across bins of 10-year period starting at 25 years is depicted in Fig. 2C. As age increased from the relatively young cohort (25–34-year-old) to the relatively old cohort (≥ 65-year-old), ΔC progressively increased from 1.53 ± 0.54 m/s to 2.34 ± 0.59 m/s with p < 0.001 (Fig. 2C). There was a consistent increment (p < 0.001) in the average value of ΔC from 25–34-year bin to 55–64-year bin (10.9%, 16.0% and 18.5%, respectively. The difference in average ΔC across 55–64-year and ≥ 64-year bins were insignificant (2.35 ± 1.06 m/s versus 2.34 ± 0.59 m/s, p = 0.96). In a multivariable model fitted for ΔC with covariates adjusted, the age was significantly associated with ΔC values (β = 0.025, p < 0.001). The association was persevered when the analysis was performed individually for males (β = 0.028, p < 0.001) and females (β = 0.036, p < 0.001). In the additional analyses with the risk factors as independent variables, while stratifying for the age, the systolic blood pressure in units of mmHg (β = 0.045, p < 0.001), diastolic blood pressure in units of mmHg (β = – 0.056, p < 0.001), high-density lipoprotein cholesterol in units of mg/dL (β = 0.084, p < 0.001), and plasma glucose in units of mg/dL (β = 0.036, p < 0.001) contributed as the most significant risk factors in determining ΔC. This trend was consistent when individual ΔC models were constructed for males and females.

### Effect of risk factors and specific MetS clusters on ΔC

As depicted in Fig. 2D, among the five risk factor groups, the I-BP group had a significantly (p < 0.001) higher difference in ΔC than the normal blood pressure group (2.02 ± 0.86 m/s versus 1.59 ± 0.54 m/s). E-BG and H-TG yielded moderate difference in ΔC from their normal groups: 1.91 ± 0.79 m/s versus 1.65 ± 0.64 m/s (p < 0.001) and 1.87 ± 0.76 m/s versus 1.76 ± 0.72 m/s (p < 0.01), respectively. Inter-group difference in ΔCs for the normal and suspected R-HDL was marginally significant (1.75 ± 0.72 m/s versus 1.83 ± 0.75 m/s, p = 0.05) while an insignificant difference was observed for the normal and suspected L-WC (1.78 ± 0.74 m/s versus 1.85 ± 0.75 m/s, p = 0.20).

**Figure 3:**
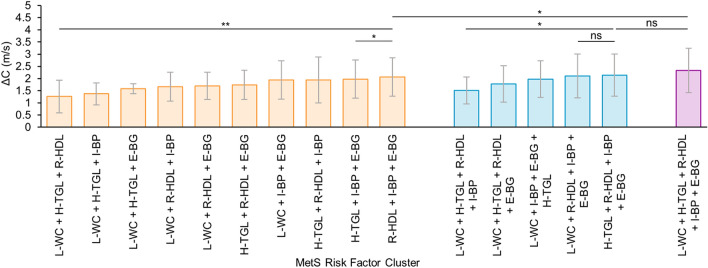
Clustering effect of multiple (3, 4, and 5) risk factors on ΔC across MetS group (N = 455) (ns p > 0.05, * p ≤ 0.05, ** p ≤ 0.001).

In compliance with earlier studies depicting the effect of specific clusters of MetS components on large artery stiffness^25,26^, the magnitude of ΔC was found to be associated with clustering of multiple risk factors (Fig. 3). Among 16 possible combinations of risk factors, the clusters comprising of R-HDL, I-BP, and E-BG was the most prominent in MetS group (43.9%) while also yielding higher ΔC values: 2.06 ± 0.77 m/s for [R-HDL + I-BP + E-BG], 2.11 ± 0.89 m/s for [L-WC + R-HDL + I-BP + E-BG] and 2.14 ± 0.86 m/s for [H-TG + R-HDL + I-BP + E-BG], and 2.33 ± 0.91 m/s for [L-WC + H-TGL + R-HDL + I-BP + E-BG]. Note also that the higher quartile of ΔC within MetS group (quartile average: 3.14 ± 0.78 m/s) comprised of 60% subjects diagnosed with clusters of R-HDL, I-BP, and E-BG compared to 32.5% in the lower quartile (quartile average: 1.11 ± 0.22 m/s). The cluster [L-WC + H-TGL + R-HDL] was sparse in MetS group (1.75%) with a ΔC of 1.25 ± 0.67 m/s. Among the clusters of four risks, [L-WC + H-TGL + R-HDL + I-BP] was infrequent (0.87% of MetS group) and yielded ΔC = 1.51 ± 0.55 m/s. The trend described retained after adjustment for covariates.

### Predictive role of ΔC in risk classification

**Figure 4:**
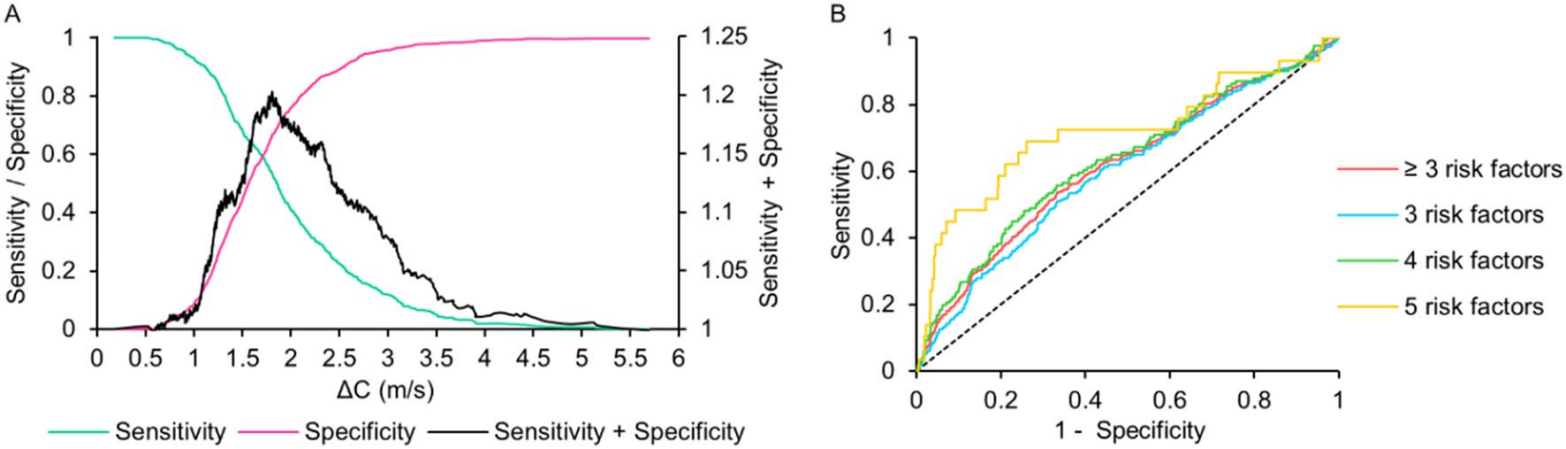
(**A**) Incremental pulse wave velocity, ΔC, versus sensitivity and specificity for the occurrence of ≥ 3 risk factors, (**B**) Receiver Operating Characteristic (ROC) curves for prediction of MetS with 3, 4, 5 or ≥ 3 risk factors.

In the logistic regression analysis after adjusting for covariates (Table 2), third and fourth quartiles of ΔC (quartile average: 1.87 ± 0.13 m/s and 2.79 ± 0.69 m/s) were associated with ~ 1.5 and ~ 2.5 times increased odds of occurrence of MetS, respectively. The plot of sensitivity versus specificity (Fig. 4A) revealed 1.81 m/s as the optimal cut-off for ΔC to predict latent clustering of three or more risk factors (manifestation of MetS). This result was also evident in the corresponding receiver-operating characteristic (ROC) curve (Fig. 4B) with an area under the curve (AUC) of 0.615 (95% CI 0.582–0.648, p < 0.001). When tested in MetS subjects with 3 risks (Fig. 4B), the optimal cut-off ΔC was 1.81 m/s with an AUC equal to 0.598 (95% CI 0.560–0.637, p < 0.001). Despite identical ΔC cut-offs, ROC curve area for ≥ 3 risk factors was higher than that of 3 risk factors. The optimal cut-off ΔC to screen MetS subjects with 4 risk factors, as revealed by the ROC curve analysis (Fig. 4B), was 1.94 m/s, with an AUC of 0.631 (95% CI 0.577–0.686, p < 0.001). Similar analyses for MetS subjects with 5 risk factors (Fig. 4B) yielded 1.97 m/s as the cut-off for ΔC with an AUC of 0.714 (95% CI 0.597–0.832, p < 0.001). ROC curve area for 5 risk factors was higher than that of the 4, 3, and ≥ 3 risk factors by 13.2%, 19.4%, and 16.1%, respectively. By considering ΔC ≥ 1.81 m/s as the probability cut-off for the occurrence of MetS (three or more risks), the sensitivity–specificity analysis yielded an odds ratio of 2.309 (95% CI 1.829–2.914) and relative risk of 1.703 (95% CI 1.47–1.973).

**Table 2:** Logistic regression model for the occurrence of MetS associated with the carotid ΔC.

Quartile	Average ΔC (m/s)	N	Odds ratio	95% confidence interval
1	1.05 ± 0.20	326	0.58	0.17–1.98
2	1.46 ± 0.10	326	0.11	0.08–0.71
3	1.87 ± 0.13	326	1.55	1.08–2.21
4	2.79 ± 0.69	325	2.47	1.12–4.37

## Discussion

The independent predictive role of arterial stiffness indices such as the carotid-femoral pulse wave velocity for adverse cardiovascular events and all-cause mortality has been well documented^27^. However, the pathophysiology that perturbs the arterial stiffness in subjects with metabolic abnormalities is yet to be fully understood^28,29^. To our knowledge, the present study is the first to demonstrate the association of ΔC with cardiometabolic risk factors and its possible predictive role for MetS incidents at a population level. Measurements obtained in this study showed that pulse wave velocity varies substantially between systole and diastole within a single cardiac cycle (ΔC > 0), which is in agreement with theoretical models^8^. The range of ΔC was consistent with recent studies exploiting the predictive role of intra-cycle variation in pulse wave velocity and its use as a therapeutic target. For instance, a population-level study on a cohort of 1776 individuals provided evidence of the association of ΔC with left ventricular mass index (LVMI), a surrogate for cardiac afterload^20^. Another study, performed on a control group and patients affected by the vascular Ehlers–Danlos syndrome (vEDS), reported a steady increment in ΔC with age classes (as also seen in Fig. 2C), and impairment in ΔC of patients with vEDS compared to healthy individuals^21^. A full discussion of similar studies using ΔC and its applications in medical instrumentation are described in detail elsewhere^8^.

Gender-specific differences in the progression of vascular stiffness and prevalence of cardiovascular diseases is a topic of growing interest^30^. Sexual dimorphism in arterial stiffening has been widely studied using the aortic (carotid-femoral) pulse wave velocity. Nevertheless, conclusions by independent researchers have been a controversial issue. For instance, while a study on 2158 healthy adults showed that carotid-femoral pulse wave velocity significantly differs between men and women (even after adjusting for covariates)^31^, no differences in its value was found between healthy asymptomatic men and women in another study^32^. Beyond the traditional aortic pulse wave velocity, a recent investigation demonstrated gender-related differences in the progression of local parameters of carotid stiffness on a cohort of 134 women and 122 men^33^. Our study provides an additional finding of the marginally higher values of ΔC for female subjects in an age-matched MetS group (Fig. 2A). This pattern of difference in ΔC concerning gender was more evident in subjects with ≥ 4 risk factors compared to the borderline or moderate-risk groups, which persisted after covariates adjustment as well. There was no significant difference in the average ΔC of age-matched Non-MetS males and females (Fig. 2A). These observations are in line with gender-specific molecular mechanisms and pathologies of vascular stiffening caused by metabolic abnormalities, sex steroid profile, and other risk factors as summarized elsewhere^34,35^.

In a variety of tests, as reported here, ΔC demonstrated a predictive ability that may have important clinical implications. The degree of change in group average ΔC was found to increase gradually depending on the number of risk factors (Fig. 2B). There was a minimum of ~ 5% (up to 14%) increase in ΔC between the immediate risk numbers. Because the results remained significant even after adjusting for covariates, an independent association of ΔC with the cardiometabolic risk factors was evident. This outcome indeed corroborates studies exploiting the effect of cardiometabolic risk factors on aortic pulse wave velocity^11,12,29,36,37^. In general, the severity of arterial stiffness and, thereby, pulse wave velocity seems to increase with the number of risk factors. Further, the logistic regression analysis showed that higher values of ΔC were significantly associated with the occurrence of three or more risks in a single entity. A close association of ΔC with four or more risk factors was also evident. According to the analysis we presented, the probability of the existence of MetS (≥ 3 risk factors) more than doubles when ΔC exceeded 1.81 m/s. This result means that subjects with ΔC ≥ 1.81 m/s were 1.7 (measure of relative risk) times more likely to develop cardiometabolic complications than subjects with ΔC < 1.81 m/s. Note that it was an initial attempt towards identifying reliable cut-off thresholds of ΔC for risk classification. The discrepancy in establishing such a cut-off depends on several factors, including the characteristics of the study population, sample size, and measurement device. Unquestionably, further evidence of randomized and controlled clinical trials is essential to establishing normative references for ΔC.

Irrespective of the Non-MetS or MetS group, I-BP was one of the most prevalent risk factors. Note that 55.2% of the total study population was suffering from hypertension (as per 2017 AHA/ACC definition^38^), in which 28.5% belonged to 25–34 years’ age group. A noticeable proportion of individuals were unaware of their Stage-1 hypertension, or, if aware, no hypertension control/management treatment was taken. In this context, it is worth reporting that questions remain regarding the blood pressure threshold at which treatment should be started, particularly in the context of the differing recommendations given by AHA/ACC^38^ and ESH/ESC^39^ guidelines. The pressure range where disagreement exists in American and European guidelines, i.e. between 130 to 140 mmHg systolic and 80 to 90 mmHg diastolic pressure, may be referred to as the ‘grey zone’. As per a recent report^40^, patients in the grey zone should undergo treatment only when there is evidence of hypertension-mediated organ damage, or they are at very high risk. The analysis conducted in this study provided a distinct grouping of patients in this grey zone, with an average (adjusted) ΔC of 1.69 ± 0.58 m/s. Note also that this was 5.6% higher than the covariates adjusted ΔC of normotensives (SBP/DBP < 130/80 mmHg) and 21.4% lower than that of chronic hypertensives (SBP/DBP ≥ 140/90 mmHg). This finding is not unexpected because the measured ΔC is a comprehensive property of an artery interrelating its stiffness, luminal dimensions, and pule propagation—biomarkers of the transmural blood pressure level. Another intriguing finding was that the sub-group of MetS subjects without I-BP risk (normotensives) showed 7.3% elevated ΔC when compared the subjects free from hypertension in the Non-MetS group. The trend persisted after adjustment for covariates. This observation is particularly important since ΔC revealed its capability to detect subtle vascular changes due to metabolic alterations in individuals without any hypertension-derived cardiac condition. In sum, besides age and chronic high blood pressure, the presence of multiple risk factors lead to perturbation in the material properties of large arteries^36,37^, thus emphasizing the need for vascular screening in routine clinical diagnostic practices.

The pathogenetic mechanisms responsible for an increase in arterial stiffness because of the conglomeration of three or more cardiometabolic risk factors in MetS has not been conclusively worked out. However, evidence has accumulated that the presence of three or more risks can impair endothelial function and contribute to an overall increase in the stiffness of large arteries^26,37,41,42^. The impact of endothelial dysfunction on arterial stiffening could be mediated through decreased production of vasoprotective NO or its bioavailability due to a rise in the circulating levels of endogenous nitric oxide synthase antagonists like ADMA^43^.

On balance, the results of this study indicate a positive association of ΔC with the cardiometabolic risk factors. Non-invasive assessment of ΔC from superficial arteries has a potential predictive role above and beyond the conventional surrogates in the diagnosis and management of MetS patients. Added consideration of ΔC is expected to yield pathophysiological particulars that are not captured by conventional measures of pulse wave velocity. It may also be noted that ΔC is a relative measure that quantifies the vessel property independent of the absolute baseline. Therefore, a comprehensive risk prediction using ΔC should be considered in conjunction with systolic and diastolic pulse wave velocity measurements.

The reported results must be interpreted within the context of the potential study limitations. A major limitation is the cross-sectional design of this study, in which only a limited number of MetS patients participated. Therefore, multi-site longitudinal studies on a larger number of Non-MetS and MetS patients would potentially provide insight into a causal relationship between ΔC and multiple risk factors. Another flaw was the lack of a nomogram for ΔC. This is a common limitation of clinical studies concerning the incremental elasticity of arteries. Although a few ΔC based studies have reported in recent years, there is no evidence on the development of a nomogram. However, we are currently putting effort into it. Since the study was conducted as a medical camp in a resource-constrained field/clinical setting, patients with carcinoma, human immunodeficiency virus, stage-IV cardiac diseases, and severe psychiatric illness were excluded, concerning their health and potential reluctance in participation by other subjects. Although the excluded patients were less than 1.5% of eligible subjects, the lack of ΔC measurements from such specific groups is a study limitation. They will be examined in imminent studies with special approvals. Finally, this work did not compare the performance of ΔC among various surrogate markers of cardiometabolic abnormalities available in the literature. This is a worthy direction for future work.

## Conclusion

The current study performed a non-invasive evaluation of ΔC that quantifies intrinsic non-linear elastic properties of large arteries. Measurements were obtained using our clinically validated ARTSENS device. Notably, the investigation was performed at a population level by collecting data from well-organized community studies. There was a significant and age-independent association between ΔC and the components of cardiometabolic risk factors, but the underlying mechanism needs to be further elucidated. As this study is the first and currently the largest one exploiting such an association, the documented information could open up avenues for active research in this domain. Thus, ΔC could be suitably incorporated into practice.

## Materials and methods

### Study population

An analytical cross-sectional study was conducted on the South Indian rural and urban population. The study on rural population was performed in a resource-constrained field setting, approved by the Institutional Human Ethics Committee (NIE/IHEC/201407-02) at the National Institute of Epidemiology, Indian Council of Medical Research, Chennai, India. 983 subjects from rural areas of Thiruvallur district participated. The study on the urban population was performed in a clinical setting, approved by the Institutional Ethics Committee (IEC-06/53/47) at Sri Ramachandra University, Chennai, India. 390 urban area subjects were recruited from Chennai, the capital city. All methods and procedures were carried out in accordance with the guidelines and regulations of both Institutional Review Boards. Inclusion criteria of participants in the study were male or female aged 20 years and above. Exclusion criteria were evidence of carcinoma, human immunodeficiency virus infection, stage-IV cardiac failure, or severe psychiatric illness. Study participants were fully informed about the study design and procedure. They were advised to come after fasting overnight on the date of the appointment. Before enrolling in the study, written informed consent was obtained in English and vernacular language. Individuals with erroneous data entry and incomplete measurements (~ 5%; 70 of the total 1373 participants) were excluded from the analysis. Data pooled from 1303 subjects drawn from the general population was analyzed.

### Evaluation of ΔC

A clinically usable expression for ΔC is derived from two fundamental biomechanics equations: (1) Hayashi’s heuristic model^44^, given in Eq. (1), which relates arterial pressure (P) and lumen diameter (D) with the pressure-independent specific stiffness of the artery (β_0_) using a reference pressure (P_R_) and corresponding diameter (D_R_); and (2) the Bramwell-Hill equation^45^, given in Eq. (2), governing the association of ‘instantaneous pulse wave velocity’ (C) with the blood mass density (ρ) and derivative of the arterial pressure to diameter (dP/dD). Discussions on the theory and validity of these fundamental relationships may be found elsewhere^8,46,47^.

1 $$\text{P}={\text{P}}_{\text{R}} {\text{e}}^{{\upbeta }_{0}\left(\frac{\text{D}}{{\text{D}}_{\text{R}}} - 1\right)}$$

2 $${\text{C}} = \sqrt {\frac{{\text{D}}}{{2\uprho }}\frac{{{\text{dP}}}}{{{\text{dD}}}}}$$

By differentiating Eq. (1) with respect to D and substituting the obtained dP/dD into Eq. (2) yields, with intermediary mathematical rearrangement, the following expression:

3 $$\text{C}=\sqrt{\frac{{\upbeta }_{0}}{{\text{D}}_{\text{R}}}\frac{\text{PD}}{2\uprho }}$$

This equation clearly shows that the velocity of the pulse wave propagating along the arterial wall inherently depends not only on its intrinsic wall property β_0_, but also on the vessel’s dimensions and transmural blood pressure level. As such, there is a change in the magnitude of C between early systole (with diastolic pressure P_D_ and diameter D_D_) and late systole (with systolic pressure P_S_ and diameter D_S_), which gives its maximum variation over a cardiac cycle defined as the incremental pulse wave velocity, ΔC. Furthermore, taking the numeric difference in C evaluated for (P_S_, D_S_) and (P_D_, D_D_) using Eq. (3), and by obtaining (β_0_/D_R_) in terms of pressure-diameter points using Eq. (1), we derive (refer Supplementary Methods online) the expression for ΔC given in Eq. (4). This formulation is more practically feasible for the assessment of ΔC from directly measurable values of systolic and diastolic blood pressure and arterial diameter.

4 $$\Delta {\text{C}} = \sqrt {\frac{{\ln \left( {{\text{P}}_{{\text{S}}} /{\text{P}}_{{\text{D}}} } \right)}}{{2\uprho \left( {{\text{D}}_{{\text{S}}} - {\text{D}}_{{\text{D}}} } \right)}}} \left( {\sqrt {{\text{P}}_{{\text{S}}} {\text{D}}_{{\text{S}}} } - \sqrt {{\text{P}}_{{\text{D}}} {\text{D}}_{{\text{D}}} } } \right)$$

### Measurements

From each participant, detailed demographic information (gathered via an interviewer-administered questionnaire), clinical data, lifestyle-related habits (smoking, tobacco chewing, and alcoholism), and body size characteristics (height, weight, waist circumference, and heart level-to-neck distance) were obtained or measured. Fasting blood specimens and urine samples were collected and transported to the accredited central laboratory for various biochemical investigations. Fasting blood glucose, low- and high-density lipoprotein cholesterol, total cholesterol, and triglycerides constituted the biomarkers of interest. Standard breakfast was provided post biosample collection. Participants were then allowed to acclimatize to the study environment in a relaxed sitting position for about ten minutes before performing the following measurements.

Two consecutive measurements of systolic (P_S_) and diastolic (P_D_) blood pressure were initially obtained using an oscillometric monitor (HEM-7101–Omron Co. Ltd., Japan). Our clinically validated ARTSENS device^48^ was then used to measure the arterial diameter (D_S_ and D_D_) from the left common carotid artery. This portable image-free ultrasound device, equipped with a 5 MHz transducer and powered by a fully automated measurement software^48^, extracted arterial diameter values over 10–15 consecutive cardiac cycles. Finally, ΔC was evaluated from the measured average pressure and diameter parameters using Eq. (4). We recommend^48–50^ for a detailed understanding of the ARTSENS technology and the procedures employing it for real-time assessment of arterial dimensions and various vascular stiffness indices.

### Statistical analysis

A full-analysis set consisting of 1303 individuals was identified. They were sorted into appropriate sub-groups according to baseline demographic or clinical characteristics. The Kolmogorov–Smirnov test was then used to examine the normality of distribution of continuous variables, which are presented as mean ± standard deviation. Categorical variables are presented as numbers and/or percentages. The correlation between two variables was evaluated using Pearson correlation coefficient (r). ANOVA was used to compare measured parameters between groups, and their distribution was illustrated using Box-and-Whisker charts. Multivariable regression models were fitted for the measured parameter and risk factors or anthropometric markers as dependent variables. Logistic regression analysis was performed to assess independent relationships between an event and the measured variable. To determine the discrimination ability and optimal cut-offs for the binary outcomes, receiver-operating characteristic (ROC) curves were plotted, and areas under the curves (AUCs) were evaluated. Odds ratio (OR) and relative risk (RR) metrics were calculated by means of the sensitivity–specificity analysis. Models were consistently adjusted for covariates (including age, gender, blood pressure, and pulse wave velocity) when relevant. In all of the tests, a p-value ≤ 0.05 denotes statistical significance.

According to the International Diabetes Federation (IDF), the National Heart, Lung, and Blood Institute (NHLBI), and the American Heart Association (AHA) definition^2^, individuals were diagnosed with metabolic syndrome—MetS, when at least three of the following five risk factors present: (1) L-WC—waist circumference ≥ 90 cm (in males) or ≥ 80 cm (in females) for Asian population; (2) H-TG—blood serum level of triglycerides ≥ 150 mg/dL; (3) R-HDL—blood serum level of high-density lipoprotein cholesterol < 40 mg/dL (in males) or < 50 mg/dL (in females); (4) I-BP—systolic blood pressure ≥ 130 mmHg and/or diastolic blood pressure ≥ 85 mmHg; (5) E-BG – glucose level ≥ 100 mg/dL in a blood sample after an overnight fast. Individuals with two or less risk factors were classified as free from metabolic syndrome—Non-MetS group.

## Supplementary Information

Supplementary Information.

## Data Availability

All relevant data and analyses are in the paper. The datasets generated during study are not publicly available due to protection of subject privacy but are available from the corresponding author upon reasonable request.
