# Supplementary material for: Association of incremental pulse wave velocity with cardiometabolic risk factors
Source: Sci Rep. 2021 Jul 29;11:15413. doi: 10.1038/s41598-021-94723-2 (PMC8322136; doi:10.1038/s41598-021-94723-2)
Supplement: Supplementary file 1 — Supplementary Information. [file 41598_2021_94723_MOESM1_ESM.pdf]

Association of Incremental Pulse Wave Velocity with Cardiometabolic Risk Factors

Nabeel P M[1, *], Dinu S Chandran[2], Prabhdeep Kaur[3], Sadagopan Thanikachalam[4], Mohanasankar Sivaprakasam[1, 5], and Jayaraj Joseph[5]

[1]Healthcare Technology Innovation Centre, IIT Madras, Chennai, 600113, India
[2]All India Institute of Medical Sciences, Department of Physiology, New Delhi, 110029, India
[3]National Institute of Epidemiology, Indian Council of Medical Research, Chennai, 600077, India
[4]Sri Ramachandra Institute of Higher Education and Research, Chennai, 600116, India
[5]Indian Institute of Technology Madras, Department of Electrical Engineering, Chennai, 600036, India
*nabeel@htic.iitm.ac.in

Supplementary Methods

Incremental pulse wave velocity in terms of arterial pressure and diameter

The functional relationship between transmural blood pressure (P) and lumen diameter of an elastic artery is nonlinear in nature in the physiological ranges [1]. In 1980, Hayashi *et al.* [2] experimentally showed that P and D could be described by an exponential function (equation (S1)). The exponent β_0, known as the pressure-independent specific stiffness of the artery, relates arterial pressure and diameter to its material property. The use of a reference standard pressure P_R and corresponding diameter D_R in the equation makes β_0 independent of arterial pressure level at the time of measurement [3].

$$P = P_R\, e^{\beta_0\left(\frac{D}{D_R} - 1\right)} \tag{S1}$$

Another well-known biomechanical model, derived by Bramwell and Hill [4], relating arterial pressure and diameter to pulse wave velocity (denoted as C in equation and expressions) is given in equation (S2). Here, ρ is the blood mass density.

$$C = \sqrt{\frac{D}{2\rho}\frac{dP}{dD}} \tag{S2}$$

The expression for the derivative of arterial pressure with respect to diameter (dP/dD), in terms of stiffness index and reference diameter, can be expressed as the following:

$$\frac{dP}{dD} = \left(\frac{\beta_0}{D_R}\right) P \tag{S3}$$

Substituting equation (S3) into (S2) yields a formulation for instantaneous value of C:

$$C = \sqrt{\frac{\beta_0}{D_R}\frac{PD}{2\rho}} \tag{S4}$$

Therefore, pulse wave velocity at peak systole (C_S) corresponding to the systolic pressure P_S and peak systolic diameter D_S is given in equation (S5). Likewise, pulse wave velocity at end-diastole (C_D) corresponding to the diastolic pressure P_D and end-diastolic diameter D_D is given in equation (S6).

$$C_S = \sqrt{\frac{\beta_0}{D_R}\frac{P_S D_S}{2\rho}} \tag{S5}$$

$$C_D = \sqrt{\frac{\beta_0}{D_R}\frac{P_D D_D}{2\rho}} \tag{S6}$$

The numeric difference between C_S and C_D, as in equation (S7), gives a general expression for the incremental pulse wave velocity (denoted as ΔC in equation and expressions).

$$\Delta C = C_S - C_D = \sqrt{\frac{1}{2\rho}\frac{\beta_0}{D_R}}\left(\sqrt{P_S D_S} - \sqrt{P_D D_D}\right) \tag{S7}$$

Assuming that ρ = 1050 kg/m^3 is constant, the above formula comprises a vessel-specific constant β_0 and an arbitrary constant D_R, in addition to the pressure-diameter values. By a simple transformation, the term β_0/D_R can be expressed in terms of directly measurable systolic (P_S, D_S) and diastolic (P_D, D_D) pressure-diameter values.

If the numeric ratio between the systolic and diastolic pressure values (P_S/P_D) is obtained using equation (S1), the following relation holds:

$$\frac{P_S}{P_D} = e^{\beta_0\left(\frac{D_S - D_D}{D_R}\right)} \tag{S8}$$

An expression for β_0/D_R can be thus derived as

$$\frac{\beta_0}{D_R} = \frac{\ln\left(\frac{P_S}{P_D}\right)}{D_S - D_D}. \tag{S9}$$

Combining equation (S9) with (S7), the expression for ΔC finally becomes

$$\Delta C = \sqrt{\frac{\ln\left({}^{P_S}/_{P_D}\right)}{2\rho(D_S - D_D)}}\left(\sqrt{P_S D_S} - \sqrt{P_D D_D}\right). \tag{S10}$$

This form of the ΔC equation contains only a known constant ρ and directly measurable pressure-diameter values, which is practically feasible and convenient in use.

Theoretically, accurate evaluation of ΔC using equation (S10) (equation (4) in the original article) requires simultaneous measurement of arterial pressure and diameter from the target site. In the context of the current study, P_S, P_D, D_S, and D_D are required to measure from a single point of the common carotid artery. Typically, the blood pressure measurements are often performed at the brachial artery level, while dimensional measurements are performed at the common carotid artery in the neck. Blood pressure measurement using a conventional bladder-type cuff from the carotid artery is not feasible. Besides the practical challenges, the accuracy of applanation tonometry in measuring the carotid pressure is still not validated and therefore, less favored [5]. Clinically validated cuffless techniques for carotid pressure measurement are also not available at present [6]. Therefore, as a common practice, the reference standard imaging ultrasound devices use blood pressure measured from the brachial artery along with the carotid arteries' geometrical measures to estimate the vascular stiffness indices [7,8]. Such an approach is widely accepted by researchers as well as used in current clinical practice. Subsequently, in this study, we evaluated ΔC using the carotid diameter (D_S and D_D) and brachial pressure (P_S and P_D), despite the known difference in systolic pressure between the brachial and carotid arteries. Further works towards the development of a cuffless device for simultaneous measurement of arterial pressure and diameter from the superficial arteries is underway [9,10], which can potentially overcome the aforesaid common limitation.

References

1. Nabeel, P. M., Raj, V. K., Joseph, J., Abhidev, V. V & Sivaprakasam, M. Local pulse wave velocity: theory, methods, advancements, and clinical applications. *IEEE Rev. Biomed. Eng.* **13**, 74–112 (2020).
2. Hayashi, K., Handa, H., Nagasawa, S., Okumura, A. & Moritake, K. Stiffness and elastic behavior of human intracranial and extracranial arteries. *J. Biomech.* **13**, 175–179, 181–184 (1980).
3. Spronck, B. *et al.* Arterial stiffness index beta and cardio-ankle vascular index inherently depend on blood pressure but can be readily corrected. *J. Hypertens.* **35**, 98–104 (2017).
4. Bramwell, J. C. & Hill, A. V. The velocity of the pulse wave in man. *R. Soc. Publ.* **93**, 298–306 (1922).
5. O'Rourke, M. F. Carotid artery tonometry: pros and cons. *Am. J. Hypertens.* **29**, 296–298 (2016).
6. Mukkamala, R. *et al.* Toward ubiquitous blood pressure monitoring via pulse transit time: theory and practice. *IEEE Trans. Biomed. Eng.* **62**, 1879–1901 (2015).
7. Vriz, O. *et al.* Comparison of sequentially measured Aloka echo-tracking one-point pulse wave velocity with SphygmoCor carotid–femoral pulse wave velocity. *SAGE Open Med.* **1**, 2050312113507563 (2013).
8. Łoboz-Rudnicka, M. *et al.* Gender-related differences in the progression of carotid stiffness with age and in the influence of risk factors on carotid stiffness. *Clin. Interv. Aging* **13**, 1183–1191 (2018).
9. Nabeel, P. M., Joseph, J., Karthik, S., Sivaprakasam, M. & Chenniappan, M. Bi-modal arterial compliance probe for calibration-free cuffless blood pressure estimation. *IEEE Trans. Biomed. Eng.* **65**, 2392–2404 (2018).
10. Joseph, J., Nabeel, P. M. & Sivaprakasam, M. Cuffless evaluation of pulse pressure with arterial compliance probe. *PLoS One* **13**, e0202480 (2018).
